# Comparison of vaginal birth outcomes in midwifery-led versus physician-led setting: A propensity score-matched analysis

**DOI:** 10.1515/med-2021-0373

**Published:** 2021-10-15

**Authors:** Ingrida Poškienė, Giedrius Vanagas, Asta Kirkilytė, Rūta Jolanta Nadišauskienė

**Affiliations:** Department of Obstetrics and Gynecology, Lithuanian University of Health Sciences, Medical Academy, Eiveniu st. 2, Kaunas, Lithuania; Institute of Pharmacoeconomics, Kaunas, Lithuania

**Keywords:** midwifery-led care, physician-led care, birth outcomes, vaginal birth

## Abstract

**Background:**

Experts in many countries are recommending a scaling up midwifery-led care as a model to improve maternal and newborn outcomes, reduce rates of unnecessary interventions, realise cost savings, and facilitate normal spontaneous vaginal birth.

**Objective:**

The aim of this study was to compare midwifery-led and obstetrician-gynaecologist-led care-related vaginal birth outcomes.

**Participants:**

Pregnant women in Kaunas city maternity care facilities.

**Methods:**

A propensity score-matched case–control study of midwifery-led versus physician-led low-risk birth outcomes. Patient characteristics and outcomes were compared between the groups. Continuous variables are presented as mean ± standard deviation and analysed using the Mann–Whitney *U* test. Categorical and binary variables are presented as frequency (percentage), and differences were analysed using the chi-square test. Analyses were conducted separately for the unmatched (before propensity score matched [PSM]) and matched (after PSM) groups.

**Results:**

After adjusting groups for propensity score, postpartum haemorrhage differences between physician-led and midwifery-led labours were significantly different (169.5 and 152.6 mL; *p* = 0.026), same for hospital stay duration (3.3 and 3.1 days, *p* = 0.042). Also, in matched population, significant differences were seen for episiotomy rates (chi^2^ = 4.8; *p* = 0.029), newborn Apgar 5 min score (9.58 and 9.76; *p* = 0.002), and pain relief (chi^2^ = 14.9; *p* = 0.002). Significant differences were seen in unmatched but not confirmed in matched population for obstetrical procedures used during labour, breastfeeding, birth induction, newborn Apgar 1 min scores, and successful vaginal birth as an overall spontaneous vaginal birth success measure.

**Conclusion:**

The midwifery-led care model showed significant differences from the physician-led care model in episiotomy rates, hospital stay duration and postpartum haemorrhage, and newborn Apgar 5 min scores. Midwifery-led care is as safe as physician-led care and does not influence the rate of successful spontaneous vaginal births.

## 1 Introduction

In the most general sense, physician-led care can be associated with the biomedical model of care, which aims to reduce the risk of maternal/foetal/infant morbidity and mortality by screening, diagnosis, and treatment of potential complications as they develop [1]. In contrast to the biomedical physician-led model, midwifery practice focuses on the normal biological processes of pregnancy, birth, and transition to parenthood [2]. It is well documented that midwifery-led care can be important to improve quality of care, outcomes, and be more efficient in the use of health care resources by reducing maternal and neonatal mortality and morbidity, reducing stillbirth and preterm birth, decreasing the number of unnecessary interventions, and improving psychosocial and public health outcomes [3]. Global health experts in many countries are recommending a scaling up midwifery-led care as a model to improve maternal and newborn outcomes, reduce rates of unnecessary interventions, realise cost savings, and facilitate normal spontaneous vaginal birth [4,5,6].

For almost half of the century, Lithuania had an obstetrician-gynaecologist-led maternity care system, in which midwives were a part of the system without autonomy in their practice. As in all antenatal and postnatal maternity care in the Soviet system, the role of midwives was diminished to the position of a doctor’s assistant. Midwives had very limited responsibilities and without possibilities to make individual clinical decisions. However, since gained independence from Soviet, there have been promising changes towards enhancing autonomy of midwifery during the last decades. In 1996 World Health Organisation (WHO) has stated: “The midwife appears to be the most appropriate and cost-effective type of health care provider to be assigned to the care of normal pregnancy and normal birth, including risk assessment and the recognition of complications” [7]. Recently, in settings with well-functioning midwifery programmes, WHO has recommended a midwifery-led continuity-of-care model, in which a known midwife or a small group of known midwives support a woman throughout the antenatal, intrapartum, and postnatal continuum [8,9].

Since 1992 Lithuania has introduced the perinatology programme and allocated midwifery services to all three levels of care. Since then, the Lithuanian Union of Midwives was active in the preparation of the legislative documentation, standardised operating procedures and regulations, and organisation of postgraduate education programmes and courses [10]. Subsequent changes from 2010 in maternity care organisation led to a new midwives’ practice, with higher midwife’s independence to provide antenatal care independently for low-risk women. And accordingly, midwifery-led care had to become essential for all low-risk births.

Currently, in Lithuania, there are two main models of care: midwiferys-led and physician-led care for the perinatal period. The first model – midwifery-led care model – is based on a normal physiological process of birth, focusing on personalised care and avoiding unnecessary medical interventions. The second model – physician-led model – is focused on safety aspects of birth. The qualification and competence of physicians permit to use more medical interventions, and also they take care of high-risk births. Accordingly, low-risk pregnant women can choose either midwiferys-led care or physician-led care. However, in some cases, low-risk pregnant women during pregnancy period can become as a high risk, and it can increase the need and use of medical interventions, changing the normal process of birth. In such cases, the obstetrician-gynaecologist will also be involved in the care process (when necessary) and medical staff will work in a team.

Obstetric interventions and procedures are the responsibilities of the obstetrician-gynaecologist. However, in Lithuania, it is regulated that some interventions can also be performed by a midwife. The midwife can perform an amniotomy, stimulate the activity by oxytocin, provide pain control, perform an episiotomy, suture the episiotomy, and provide active placental period. In the event of any complications until the arrival of the physician, midwife on her own may perform the following procedures: initiate the woman’s resuscitation; provide assistance in cases of breach presentation, umbilical cord prolapse, and foetal shoulder dystocia; if necessary, use a vacuum extractor; examine the uterus by hand and remove the placenta; and stop the bleeding from uterus by applying bimanual uterine pressure and squeezing the aorta, if necessary.

The aim of this study was to compare midwifery-led and obstetrician-gynaecologist-led care-related vaginal birth outcomes.

## 2 Methods

### 2.1 Study design

A propensity score-matched case–control study of midwifery-led versus physician-led birth outcomes. The use of propensity score allows us to compare midwifery-led and physician-led birth by matching the birth risk and other clinical characteristics between groups and minimise the potential for selection bias.

### 2.2 Setting

This study was conducted in Kaunas city maternity care units with obstetric care. We studied low-risk women with singleton pregnancies who were supervised and gave vaginal birth beyond 37 weeks of gestation.

Kaunas city has all three levels of health care facilities: two small maternities serving for low-risk births and one multiprofile regional hospital for low-risk births and births from 34 weeks for women with risk factors, and one Perinatology Center was available with all levels of care, including neonatology and intensive care units.

### 2.3 Study participants

Pregnant women in Kaunas city maternity care facilities.

### 2.4 Case definition

A study group was composed according to low-risk pregnancy attendant: midwifery-led versus physician-led low-risk pregnancies.

A low-risk pregnancy is considered for a healthy woman before pregnancy, who did not develop any diseases during pregnancy, pregnancy without complications, foetus developed without complications, and no premature birth. At first visit, the birth risk is assessed. For a low-risk birth, the midwifery-led care is proposed first, but if the pregnant woman did not agree, physician-led care option is also available. If the risk of birth changes during the antenatal period and becomes high, the obstetrician-gynaecologist will also be involved in the care process and medical staff will work in a team.

### 2.5 Selection of controls

Controls were women who were supervised and had childbirth led by physician. Selection of controls was based on the propensity score matching from a pool of physician-led births.

### 2.6 Study size

Total unmatched population was 1,848 singleton births, 1,664 led by physicians, and 184 led by midwives, and the matched population came to 174 births in each group (1:1 matching).

### 2.7 Variables

Sociodemographic variables such as age, weight, living place, pregnancy, birth number, and newborn weight were obtained from medical records.

Data on outcome variables such as postpartum haemorrhage, hospital stay duration, obstetrical procedures as uterine revision, instrumental termination of labour, perineal tears, birth induction, pain relief methods, newborn Apgar 1 and 5 min scores, and breastfeeding initiation were collected from medical records.

Successful spontaneous vaginal birth (i.e. normal birth) was assessed as birth in which labour starts spontaneously, progresses spontaneously, and gives childbirth spontaneously without any intervention [11,12].

### 2.8 Data analysis and statistical methods

We used propensity score-matched (PSM) pairs analysis to optimise the balance of baseline covariates between groups and minimise a potential bias and confounding arising from a selection of study participants. We used a one-to-one genetic matching algorithm (Rgenoud and Matching libraries in *R* software V.3.5.3 – Free Software Foundation s GNU General Public License, developed at Bell Laboratories (formerly AT&T, now Lucent Technologies) by John Chambers and colleagues.) without replacement with a caliper of 0.25 to get the best balance of the groups for potentially confounding variables showing significant differences in unmatched population. Matching was performed on following potentially confounding variables: maternal age, body mass index (BMI), pregnancy number, birth number, and birth-risk evaluation. We used effect size and significance testing to assess the balance of the covariates after matching.

Patient characteristics and outcomes were compared between the groups. Continuous variables are presented as mean ± standard deviation (SD) and analysed using the Mann–Whitney *U* test. Categorical and binary variables are presented as frequency (percentage), and differences were analysed using the chi-square test. Analyses were conducted separately for the unmatched (before PSM) and matched (after PSM) groups. Statistical data analysis was two tailed and performed using the JASP V.0.10.2. The statistical difference between the groups was considered significant when *p* < 0.05.

### 2.9 Ethical and data protection considerations

The study protocol was reviewed and approved by the Biomedical Research Ethics Committee of Kaunas region (No. BE-2-33). There was no direct contact with births and neonates during data collection and analysis. No maternal and neonatal personally identifiable information was included in the research data; therefore, the Biomedical Research Ethics Committee of Kaunas region exempted the informed consent form usage.

## 3 Results

The study data were available for 1,848 singleton low-risk births from Kaunas city maternity care units with obstetric care. Of all number of births, there were 184 births led by midwives and 1,664 births led by obstetrician-gynaecologists. Of all total 1,848 births, 348 were matched 1:1 by making each group of 174 led by midwives and births led by obstetrician-gynaecologists. Tables 1 and 2 summarise sociodemographic and systemic disorders characteristics of study participants in matched and unmatched cohorts. The matched data also indicated an overall lower difference on potential confounding variables and almost perfect match on controlled variables during the matching. The matching process improved balance of systemic disorders and sociodemographic characteristics between these groups.

**Table 1:** Sociodemographic characteristics of study participants in the unmatched and matched study populations

	Unmatched population (*N* = 1,848)			Matched population (*N* = 348)		
	Physician (*N* = 1,664)	Midwife (*N* = 184)	*p* value	Physician (*N* = 174)	Midwife (*N* = 174)	*p* value
Maternal age (years)	28.6 ± 4.1	28.9 ± 0.5	0.466	28.8 ± 3.9	28.8 ± 3.9	0.980
Maternal BMI	27.8 ± 4.1	27.0 ± 3.5	0.035	27.2 ± 3.8	27.0 ± 3.6	0.721
Maternal weight (kg)	78.4 ± 12.3	76.4 ± 10.9	0.075	76.6 ± 11.3	76.3 ± 10.8	0.922
Newborn weight (kg)	3.54 ± 0.30	3.48 ± 0.35	0.081	3.49 ± 0.35	3.47 ± 0.35	0.321
Primiparas	704 (42.3)	46 (25.0)	<0.001	44 (25.3)	44 (25.3)	1.000
Birth no.	1.6 ± 0.6	1.8 ± 0.6	<0.001	1.8 ± 0.5	1.8 ± 0.5	1.000
Pregnancy no.	1.9 ± 0.9	2.1 ± 0.8	<0.001	2.0 ± 0.7	2.0 ± 0.7	1.000
Pregnancy duration (days)	39.4 ± 1.0	39.2 ± 1.1	0.105	39.4 ± 0.9	39.2 ± 1.0	0.223
Previous pregnancies						
None	610 (36.7)	44 (23.9)	<0.001	42 (24.2)	44 (25.3)	0.968
Normal	613 (36.8)	92 (50.0)		90 (51.7)	89 (51.1)	
Complicated	441 (26.5)	48 (26.1)		42 (24.1)	41 (23.6)	
Living place = city	1,166 (70.1)	133 (72.3)	0.534	122 (70.1)	125 (71.8)	0.723

Note. Continuous variables were reported as mean ± SD, and categorical variables were reported as counts and percentages. *P*-value was determined by the Mann–Whitney *U* test for continuous variables and chi^2^ test for categorical and binary variables.

**Table 2:** Systemic disorders of study participants in the unmatched and matched study populations

	Unmatched population (*N* = 1,848)			Matched population (*N* = 348)		
	Physician (*N* = 1,664)	Midwife (*N* = 184)	*p* value	Physician (*N* = 174)	Midwife (*N* = 174)	*p* value
Hypertension	666 (40.0)	67 (36.4)	0.342	67 (38.5)	65 (37.4)	0.825
Allergy	240 (14.4)	27 (14.7)	0.927	24 (13.8)	26 (14.9)	0.760
Heart disease	84 (5.0)	7 (3.8)	0.459	5 (2.9)	7 (4.0)	0.557
Endocrinological diseases	90 (5.4)	12 (6.5)	0.530	10 (5.7)	11 (6.3)	0.822
Tuberculosis	47 (2.8)	3 (1.6)	0.343	4 (2.3)	3 (1.7)	0.703
Urinary tract diseases	252 (15.1)	26 (14.1)	0.715	24 (13.8)	23 (13.2)	0.875
Preeclampsia	17 (1.0)	0 (0.0)	0.168	1 (0.6)	0 (0.0)	0.317
Viral infection	117 (7.0)	7(3.8)	0.097	11 (6.3)	6 (3.5)	0.214
Epilepsy	11 (0.7)	0 (0.0)	0.269	1 (0.6)	0 (0.0)	0.317
Infection in the mouth	55 (3.3)	11 (5.9)	0.064	7 (4.0)	10 (5.7)	0.456

Note. Continuous variables were reported as mean ± SD, and categorical variables were reported as counts and percentages. *P*-value was determined by the Mann–Whitney *U* test for continuous variables and chi^2^ test for categorical and binary variables.

Table 3 shows comparison for each of investigated birth outcome in the unmatched and matched study populations. Postpartum haemorrhage differences between physician-led and midwifery-led labour were significant in both unmatched and matched populations (*p* = 0.007 and 0.026), same for hospital stay duration (*p* = 0.001 and 0.042), episiotomy (*p* = <0.001 and 0.029), newborn Apgar 5 (*p* = 0.001 and 0.002) and pain relief (*p* < 0.001 and *p* = 0.002).

**Table 3:** Comparison of birth outcomes and services in the unmatched and matched study populations

Outcomes	Unmatched population (*N* = 1,848)			Matched population (*N* = 348)		
	Physician (*N* = 1,664)	Midwife (*N* = 184)	*p* value	Physician (*N* = 174)	Midwife (*N* = 174)	*p* value
Postpartum haemorrhage (mL)	169.9 ± 99.6	152.4 ± 70.4	0.007	169.5 ± 90.1	152.6 ± 71.4	0.026
Hospital stay duration (days)	3.4 ± 1.4	3.1 ± 0.9	0.001	3.3 ± 1.4	3.1 ± 0.9	0.042
Obstetrical procedures						
None	1,589 (95.5)	183 (99.5)	0.036	167 (96.0)	173 (99.4)	0.086
Revision	55 (3.3)	1 (0.5)		4 (2.3)	1 (0.6)	
Instrumental	20 (1.2)	0 (0)		3 (1.7)	0 (0.0)	
Perineal tears						
First- and second-degree tears	316 (19.0)	45 (24.5)	0.926	45 (25.9)	42 (24.1)	0.079
Third- and fourth-degree tears	1 (0.1)	0 (0.0)		0 (0.0)	0 (0.0)	
Genital tears	109 (5.4)	15 (8.2)		6 (3.4)	14 (8.0)	
Episiotomy	402 (24.2)	17 (9.2)	<0.001	31 (17.8)	16 (9.2)	0.029
Intact perineum	836 (50.2)	107 (58.2)	0.01	92 (52.9)	102 (58.6)	0.473
Birth induction						
None	837 (50.3)	115 (62.5)	<0.001	101 (58.0)	108 (62.1)	0.199
Rupture the amniotic fluid	237 (14.2)	39 (21.2)		27 (15.5)	37 (21.3)	
Oxytocinum	380 (22.8)	22 (12.0)		33 (19.0)	22 (12.6)	
Misoprostolum	29 (1.7)	1 (0.5)		1 (0.6)	1 (0.6)	
Other	181 (10.9)	7 (3.8)		12 (6.9)	6 (3.4)	
Apgar 1 min. score	9.07 (0.7)	9.22 (0.5)	0.017	9.08 (0.8)	9.23 (0.5)	0.152
Apgar 5 min. score	9.61 (0.6)	9.76 (0.5)	0.001	9.58 (0.6)	9.76 (0.5)	0.002
Pain relief						
None	987 (59.3)	130 (70.7)	<0.001	121 (69.5)	122 (70.1)	0.002
NO_2_	76 (4.6)	21 (11.4)		4 (2.3)	20 (11.5)	
Spinal-epidural	581 (34.9)	33 (17.9)		48 (27.6)	32 (18.4)	
Other	20 (1.2)	0 (0.0)		1 (0.6)	0 (0.0)	
Breastfeeding	1,233 (89.7)	174 (95.1)	0.02	126 (92.0)	164 (94.8)	0.314
Successful vaginal birth	325 (19.5)	57 (31.0)	<0.001	46 (26.4)	54 (31.0)	0.343

Note: Continuous variables were reported as mean ± SD, and categorical variables were reported as counts and percentages. *P*-value was determined by Mann–Whitney *U* test for continuous variables and chi^2^ test for categorical and binary variables.

Significant differences were seen in unmatched but not confirmed in matched population for obstetrical procedures used during labour, breastfeeding, birth induction, newborn Apgar 1 and successful vaginal birth as overall spontaneous vaginal birth success measure.

## 4 Discussion

Midwifery-led care was associated with several benefits for mothers and babies and have almost no identified adverse effects in randomized controlled trials [13]. The main benefits of midwifery-led care are a reduction in the use of regional analgesia, with fewer episiotomies or instrumental births. Studies also reported that midwifery-led care can increase the woman’s chance of being individually cared and the chance of feeling in control during labour, having a spontaneous vaginal birth and initiating breastfeeding after birth. However, there was no difference in caesarean birth rates [13]. In this retrospective study, we analysed midwifery-led and obstetrician-gynaecologist-led care low-risk spontaneous vaginal birth outcomes.

### 4.1 Key results and its interpretation

Our findings are partially in line with other studies in this field although the designs of these studies were different. Our study showed significantly lower postpartum haemorrhage (*p* = 0.007 and 0.026) and shorter hospital stay duration (*p* = 0.001 and 0.042) in both unmatched and matched populations were seen for births led by midwife. A review of clinical trials was also concluded that midwifery-led care was superior to other forms of care and was more likely to have a shorter length of hospital stay [13]. However, there is no agreement on postpartum haemorrhage. Recent retrospective cohort study concluded that women in midwifery-led care had increased the odds of postpartum haemorrhage; however, it did not remain significant in the propensity score analysis [14]. Some other study reported lower rates of postpartum haemorrhage [15]. The studies reported that incidence and severity of postpartum bleeding are related to more frequent use of obstetric interventions [16,17,18,19], and postpartum haemorrhage can be increased by twin births (OR 6.8), retained placenta (OR 3.9) and inductions of labour (OR 2.2), birthweight >4,000 g (OR 2.0), and sphincter rupture (OR 1.6) [13]. Our data show that the midwifery-led births were less medicalised by pain-relief methods, birth induction, and obstetric interventions. It is possible that midwifery-led care provides more personalised and individual needs and preferences-focused care, which leads to a lower rate of clinical interventions. Previous studies did not demonstrate significant differences in Apgar scores between midwifery-led care and other models [15,20,21]; however, our study results demonstrated higher Apgar 5 (*p* = 0.001 and 0.002) scores were recorded for midwifery-led births; however, Apgar 1 score differences were not significant.

The prospective birthplace in England study group was analysed for a composite poor neonatal outcome among low-risk women and found that the proportion of women with a successful spontaneous vaginal birth as “normal birth” (birth without induction of labour, epidural or spinal analgesia, general anaesthesia, forceps or ventouse birth, caesarean section or episiotomy) varied from 58% of births in physician-led units to 76% in alongside midwifery units; the adjusted odds of having a “normal birth” were significantly higher in non-obstetric unit setting. Other maternal outcomes (third- or fourth-degree perineal trauma, maternal blood transfusion, and maternal admission to higher level care) were generally lower for planned births in midwifery units [11,22]. Our study did not confirm higher rates of “normal birth” as composite outcome of successful spontaneous vaginal birth in midwifery-led care by PSM analysis, and the differences were significant only in unmatched population. Similar results were for breastfeeding initiation after birth, despite some studies argued better outcomes for midwifery-led care births.

### 4.2 Strengths and weaknesses

The main weakness of the study is that due to low sample size in matched analysis, we did not examine the inter-relationships between different predictors of outcomes across physician-led and midwifery-led care models. Although we identified certain outcomes were different across settings, we did not examine why these changes occur. Alternatively, there are sufficient number of previous studies explaining such inter-relationships between outcomes and potential factors.

Usually with case–control studies, there are concerns about the problem of selection bias when comparing different practices and potential that, in settings like ours, there is a possibility to choose both models of care in the same unit, the women who were selected or allocated to midwifery-led care might be healthier and at lower risk. Thus, the main strength of our study is the fact that we created comparable groups through group matching, thereby attempting to reduce bias due to confounding variables such as maternal age, BMI, pregnancy number, birth number, and birth risk evaluation score. Due to the use of genetic matching algorithm, we improved balance of clinical and sociodemographic characteristics between the groups not only on matched variables.

### 4.3 Generalisability

Our study showed promising results for enhancing autonomy of midwifery-led care and autonomous work at labour wards for low-risk women in Lithuanian maternity health care system. The clinical practice should be more focused on the normal biological processes of pregnancy, birth, and transition to parenthood and further reduction of unnecessary medicalisation of birth.

## 5 Conclusion

Midwifery-led care showed significant differences from physician-led care model in episiotomy rates, hospital stay duration, postpartum haemorrhage, and newborn Apgar 5 min scores. Midwifery-led care is as safe as physician-led care and care model selection does not influence rate of successful spontaneous vaginal births.

## References

[1] Bartuseviciene E, Kacerauskiene J, Bartusevicius A, Paulionyte M, Nadisauskiene RJ, Kliucinskas M, et al. Comparison of midwife-led and obstetrician-led care in Lithuania: a retrospective cohort study. Midwifery. 2018 Oct 1;65:67–71. 10.1016/j.midw.2018.06.017 29980361

[2] Bateman B, Berman M, Riley L, Leffert L. The epidemiology of postpartum hemorrhage in a large, nationwide sample of deliveries. Anesth Analg. 2010 May;110(5):1368–73. 10.1213/ANE.0b013e3181d74898 20237047

[3] Benatar S, Garrett AB, Howell E, Palmer A. Midwifery care at a freestanding birth center: a safe and effective alternative to conventional maternity care. Health Serv Res. 2013;48(5):1750–68. 10.1111/1475-6773.12061 PMC3796112 23586867

[4] Fein A, Wen T, Wright JD, Goffman D, D’Alton ME, Attenello FJ, et al. Postpartum hemorrhage and risk for postpartum readmission. J Matern Fetal Neonatal Med. 2019 Mar 28;34(2):1–8. 10.1080/14767058.2019.1601697 PMC7135873 30919702

[5] Halpern S. SOGC joint policy statement on normal childbirth. J Obstet Gynaecol Can. 2009 Jul 1;31(7):602. 10.1016/S1701-2163(16)34236-0 19771676

[6] Hatem M, Sandall J, Devane D, Soltani H, Gates S. Midwife-led versus other models of care for childbearing women. Cochrane Database Syst Rev. 2008 Oct 8;4:CD004667. 10.1002/14651858.CD004667.pub2 18843666

[7] World Health Organization. Care in Normal Birth: a practical guide; 1996. 9271979

[8] World Health Organization. WHO recommendations on antenatal care for a positive pregnancy experience. Luxemburg; 2016. 28079998

[9] World Health Organization. WHO recommendations: intrapartum care for a positive childbirth experience. Geneva; 2018. 30070803

[10] Homer CSE, Friberg IK, Dias MAB, Hoope-Bender P, ten, Sandall J, Speciale AM, et al. The projected effect of scaling up midwifery. Lancet. 2014 Sep 20;384(9948):1146–57. 10.1016/S0140-6736(14)60790-X 24965814

[11] Jackson DJ, Lang JM, Swartz WH, Ganiats TG, Fullerton J, Ecker J, et al. Outcomes, safety, and resource utilization in a collaborative care birth center program compared with traditional physician-based perinatal care. Am J Public Health. 2003 Jun 1;93(6):999–1006. 10.2105/ajph.93.6.999 PMC1447883 12773368

[12] Knight M, Callaghan WM, Berg C, Alexander S, Bouvier-Colle M-H, Ford JB, et al. Trends in postpartum hemorrhage in high resource countries: a review and recommendations from the international postpartum hemorrhage collaborative group. BMC Pregnancy Childbirth. 2009 Nov 27;9:55. 10.1186/1471-2393-9-55 PMC2790440 19943928

[13] Lerberghe WV, Matthews Z, Achadi E, Ancona C, Campbell J, Channon A, et al. Country experience with strengthening of health systems and deployment of midwives in countries with high maternal mortality. Lancet. 2014 Sep 27;384(9949):1215–25. 10.1016/S0140-6736(14)60919-3 24965819

[14] Overgaard C, Møller AM, Fenger-Grøn M, Knudsen LB, Sandall J. Freestanding midwifery unit versus obstetric unit: a matched cohort study of outcomes in low-risk women. BMJ Open. 2011 Jan 1;1(2):e000262. 10.1136/bmjopen-2011-000262 PMC3191606 22021892

[15] Renfrew MJ, McFadden A, Bastos MH, Campbell J, Channon AA, Cheung NF, et al. Midwifery and quality care: findings from a new evidence-informed framework for maternal and newborn care. Lancet. 2014 Sep 20;384(9948):1129–45. 10.1016/S0140-6736(14)60789-3 24965816

[16] Rossen J, Økland I, Nilsen OB, Eggebø TM. Is there an increase of postpartum hemorrhage, and is severe hemorrhage associated with more frequent use of obstetric interventions? Acta Obstet Gynecol Scand. 2010;89(10):1248–55. 10.3109/00016349.2010.514324 20809871

[17] ten Hoope-Bender P, de Bernis L, Campbell J, Downe S, Fauveau V, Fogstad H, et al. Improvement of maternal and newborn health through midwifery. Lancet. 2014 Sep 27;384(9949):1226–35. 10.1016/S0140-6736(14)60930-2 24965818

[18] The Maternity Care Working Party. Making normal birth a reality – Consensus Statement; 2007. p. 8.

[19] Weisband YL, Klebanoff M, Gallo MF, Shoben A, Norris AH. Birth outcomes of women using a midwife versus women using a physician for prenatal care. J Midwifery Womens Health. 2018 Jul;63(4):399–409. 10.1111/jmwh.12750 29944777

[20] Werkmeister G, Jokinen M, Mahmood T, Newburn M. Making normal labour and birth a reality – developing a multi disciplinary consensus. Midwifery. 2008 Sep;24(3):256–9. 10.1016/j.midw.2008.06.001 18672321

[21] van Teijlingen E. A critical analysis of the medical model as used in the study of pregnancy and childbirth. Sociol Res Online. 2005 Jul 1;10(2):63–77.

[22] Vedam S, Stoll K, MacDorman M, Declercq E, Cramer R, Cheyney M, et al. Mapping integration of midwives across the United States: Impact on access, equity, and outcomes. PLoS One. 2018 Feb 21;13(2):e0192523. 10.1371/journal.pone.0192523 PMC5821332 29466389

